# A Review of White Spot Lesions: Development and Treatment with Resin Infiltration

**DOI:** 10.3390/dj12120375

**Published:** 2024-11-22

**Authors:** Alexandra Maria Prada, Georgiana Ioana Potra Cicalău, Gabriela Ciavoi

**Affiliations:** Department of Dental Medicine, Faculty of Medicine and Pharmacy, University of Oradea, 410073 Oradea, Romania; aale.prada@gmail.com (A.M.P.); gciavoi@uoradea.ro (G.C.)

**Keywords:** minimally invasive, resin infiltration, white spot lesion treatment, orthodontic white spot lesion, white spot lesion development

## Abstract

White spot lesions (WSLs) are demineralized lesions of the enamel that form in the presence of bacterial plaque, affecting the aesthetics by modifying the refractive index of the enamel, giving the characteristic “chalky” aspect. They have various causes, including fixed orthodontic treatments, improper hygiene, fluorosis and genetic factors. Background/Objectives: Considering the latest need for dental aesthetics and the popularization of fixed orthodontic treatments, the need to effectively treat WSLs has increased. The objective of this research is to review the development of WSLs and their treatment with resin infiltration. Methods: The PubMed, Web of Science, Scopus and Google Scholar databases were searched for relevant reviews and studies. Out of all, 56 were included in this research. Results: Prophylactic measures, such as fluorized toothpaste and varnishes, have limited results. Standard caries treatment is too invasive as it removes too much healthy enamel for obturation retentivity. The resin infiltration resin process does not require drilling or tooth structure loss, making it a painless and minimally invasive treatment. The resin used has a refractive index comparable to that of healthy enamel, consequently restoring aesthetics and ensuring the prevention of caries evolvement. The treatment involves five important steps: prophylaxis, acid demineralization, alcohol drying, resin infiltration and UV light curing. Depending on the clinical case, the demineralization and drying steps may need to be repeated. Conclusions: Infiltrations with resin are painless and well tolerated by patients. Out of all minimally invasive treatments, they have an immediate satisfactory outcome, with results stable for a minimum of 45 months.

## 1. Introduction

G.V. Black described white spot lesions (WSLs) as “occasional white or ashy grey spots that were small and covered with the ordinary glazed surface of the enamel so that an exploring tine will glide over them the same as over the perfect enamel” [1].

WSLs are incipient caries caused by the acidic aggression of carious biofilm. The development of dental caries has a strong correlation with tooth structure and dental plaque [2]. The demineralization of teeth undergoing fixed orthodontic treatment affects the enamel structure, debilitating the teeth’s resistance to aggressions. In addition to that, fixed appliances badly influence the possibility of correct oral hygiene.

The formation of dental plaque secondary to poor oral hygiene strongly influences the formation of dental caries. Other factors that sustain the formation of caries include diet, mineral composition of the saliva and genetic factors [3,4].

The chalky appearance of WSLs is caused by the difference between the refractive index (RI) of healthy and demineralized enamel. The RI of sound enamel is 1.66. The RI of WSLs is dependent on the hydration of the lesion. Saliva has an RI of 1.33 within the lesion, whereas air has an RI of 1.00. The difference between RIs makes dehydrated WSLs appear more evident, giving the lesion the characteristic look of a milky white and opaque spot [3,4].

Due to the particular features of WSLs and the need to treat them as minimally invasive as possible, different treatments and products have been developed.

Techniques such as microabrasion and remineralization with fluoride or casein phosphopeptide were developed but resulted in minimal results [4] or tooth substance loss in the case of microabrasion [5]. Studies showed that better outcomes were obtained through resin infiltration [6].

Resin infiltration treatment uses a low-viscosity resin that penetrates the lesion [4]. The resin used has refractive properties similar to those of the healthy enamel, thus restoring the aspect and colour [1]. 

The procedure consists of etching, drying and infiltration steps [7]. Depending on the clinical case, the first two steps can be repeated. Some manufacturers encourage at least two etching sessions, each followed by drying steps.

This treatment can restore aesthetics and arrest the carious progression in one simple procedure [6,7,8].

Aim and objectives: This study aims to describe the development of white spot lesions and to present their treatment with resin infiltration. The review of the procedure routine, the comparison between the results of clinical studies and emphasis of the advantages and disadvantages of this procedure are critical for a better understanding.

## 2. Materials and Methods

For the theoretical part of this review, PubMed, Web of Science, Scopus and Google Scholar databases were searched for relevant scientific articles and studies. The search was conducted with the following keywords: “orthodontic white spot lesion”, “white spot lesion development” and “white spot lesion treatment”.

**Figure 1:**
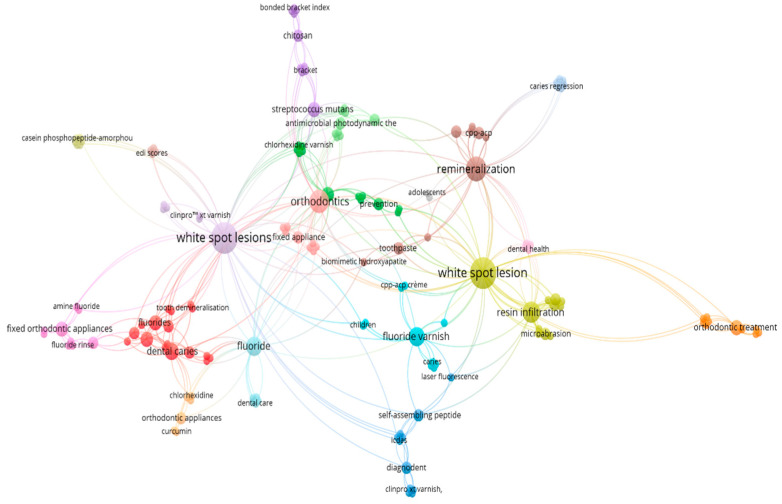
Network visualization of the white spot lesion infiltration treatment.

There were 365 keywords identified across all articles. All irrelevant keywords, including age and gender, were eliminated. The investigation revealed that the most frequently used terms were “white spot lesions”, “orthodontics”, “remineralization” and “fluoride varnish.” A distinct colour is used to represent each cluster (Figure 1).

The nodes represent keywords found in articles, and the lines represent the link between them. The size of the nodes is directly proportional to the number of uses. This figure is the author’s own creation.

**Figure 2:**
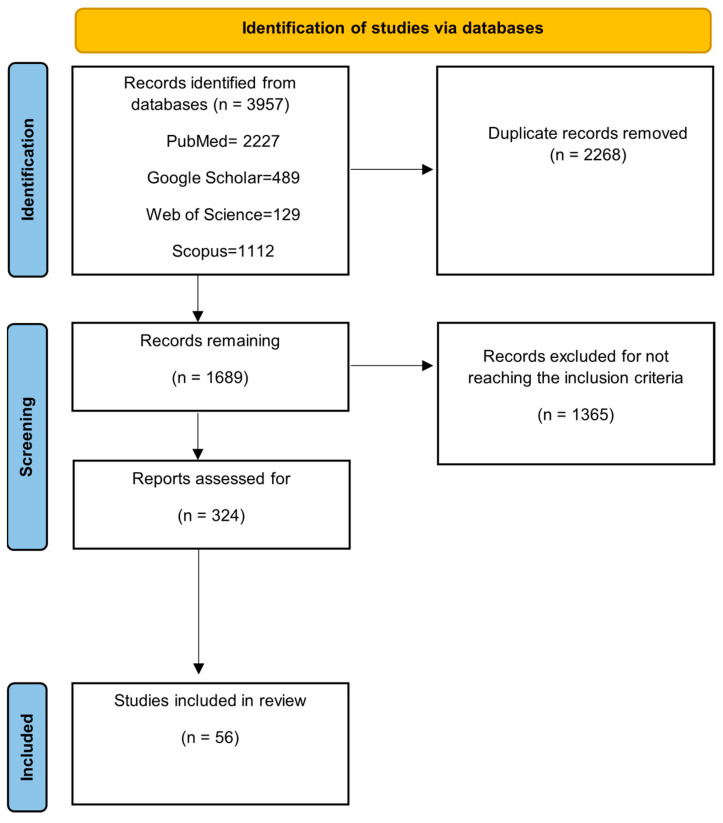
Flow chart of the article selection process.

There was a total of 3957 results. The filters applied to the search were (1) publication date no older than 2010, (2) only articles in English and (3) both trials and reviews were screened. Only publications after 2010 were pursued in the election process for present relevance and accuracy. After the filters were applied and duplicates were removed, only 324 results remained. Out of those remaining publications, after a full article review, 56 were selected for the present review (Figure 2).

The selection and exclusion process can be observed in the following table (Table 1).

**Figure 3:**
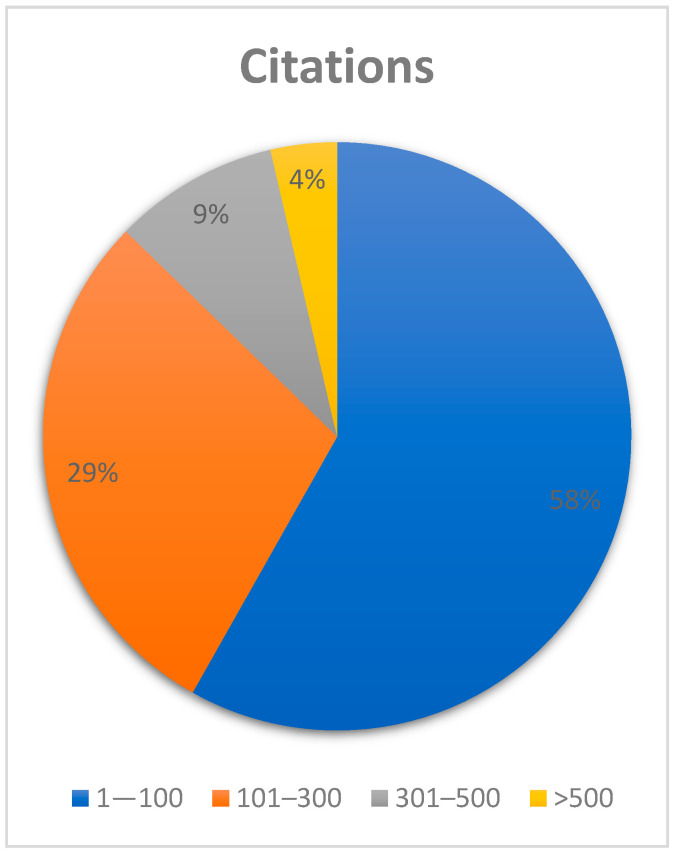
Chart illustrating the percentage distribution of the articles included according to the number of citations in the literature.

To validate the value of this research, bibliometric data were included. Both the number of citations and the impact factor of the journal of publication are depicted in the following charts. Out of 56 articles researched, 32 had between 1 and 100 citations, 16 had between 101 and 300 citations, 5 had between 301 and 500 citations and 2 had over 500 citations (Figure 3).

**Figure 4:**
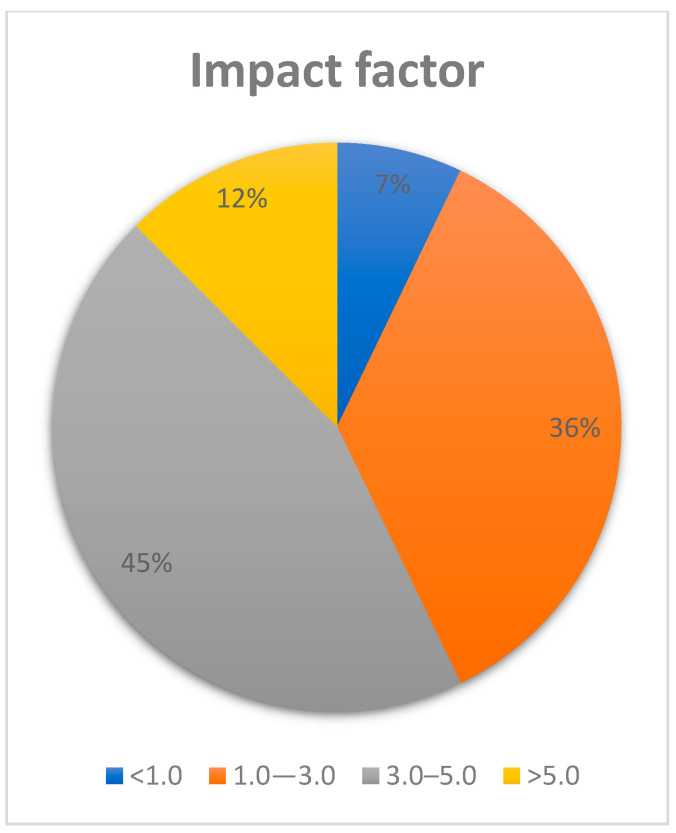
Chart illustrating the distribution of articles based on the journals’ impact factor.

Most articles researched were published in journals such as “Angle Orthodontist”, “European Journal of dentistry” and “Progress in Orthodontics”. Out of 43 articles included in this review, 4 were published in journals with an impact factor less than 1, 20 were published in journals with an impact factor between 1.0 and 3.0, 25 were published in journals with an impact factor between 3.0 and 5.0 and 7 articles were 3 of 10 published in journals with an impact factor over 5.0 (Figure 4).

**Figure 5:**
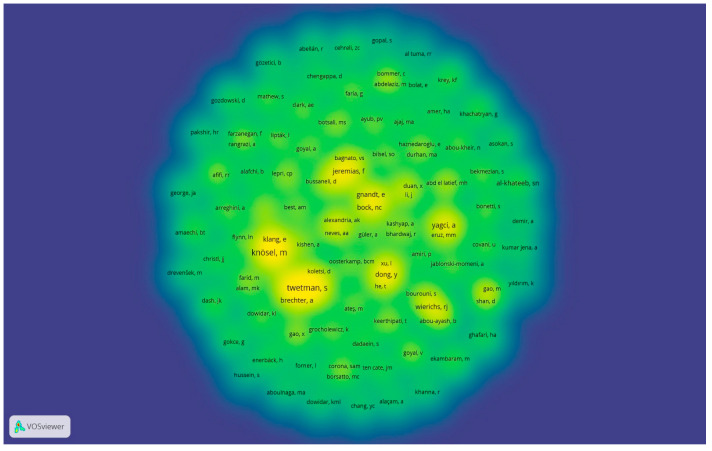
Coauthorship analysis of authors. Network visualization of the authors. The figure is the author’s own creation.

A total of 433 authors were identified and included in the coauthorship analysis. Not all authors were connected; only 17 connections between the authors were found. The difference between the size and colour of the clusters represents the density of documents (Figure 5).

For Section 3.5 of this article, the PubMed database was researched with the following keywords: “white spot lesion treatment” and “white spot lesion infiltration treatment”. There was a total of 1129 articles. The exclusion criteria used were (1) publication date no older than ten years, (2) article type: clinical trial or randomized controlled trial and (3) the article language was English. After the exclusion criteria were applied, 118 studies remained, of which 17 were duplicates. Only trials on patients were selected for this section of the article. For the infiltration technique documentation, only post-orthodontic treatment patient trials were screened, of which seven were used in this review. Other methods of WSL treatment were reviewed, such as fluoride varnish, CPP-ACP and P11-4 to corroborate the better treatment for such lesions. Both patients undergoing and post-orthodontic treatment trials were studied, as well as patients with initial carious lesions due to poor oral hygiene trials were screened, out of which six were included in this review.

## 3. Results

### 3.1. Development of White Spot Lesions

The human oral cavity is populated by a diverse microbiome, unique to other microbiomes of the human body. The oral cavity consists of both hard tissue and soft tissue, therefore creating two different ecological niches for two distinctive biomes [9]. Dental plaque is one of the distinctive biomes; it consists of diverse microorganisms found on the tooth surface, embedded in a matrix formed of extracellular polymeric substances [10,11]. In vitro studies show it is formed and matured in 24 to 72 h [11]. It is composed of Gram-negative microorganisms such as Actinomycens, Fusobacterium and Prevotella species, as well as Gram-positive streptococci such as S. sanguinis, S. oralis, S. mitis and Neisseria species [11,12,13]. This, in correlation with a diet rich in sugars and carbohydrates, promotes acidic metabolite production and acidogenic microorganism reproduction [14].

The development of caries is a dynamic and complex process. It consists of demineralization and remineralization processes, alternating in a short period of time. As acids build up at the enamel–plaque interface, the pH lowers, and demineralization begins [2]. When demineralization persists and is favoured by the extended presence of plaque and a debilitated enamel, and when the remineralization properties of the saliva are inefficient, a porous lesion occurs that constitutes the WSLs.

The prevalence of WSLs varies widely due to a range of factors, such as diet, genetic predisposition to caries or dental hard tissue defects. Due to the broad array of possible causes, we will limit this review to the prevalence of WSLs in fixed orthodontic treatment. Before orthodontic treatment, WSLs were found in 15.5% to 40% of patients [15], and 45,8% to 68.4% had or developed WSLs during treatment [16]. The prevalence of post-treatment goes as high as 97% [17]. WSLs appear as early as two weeks after the formation of dental plaque [1,18], but most lesions appear within the first month of treatment [15,16,19]. The shape is influenced by the distribution of plaque and the direction of enamel prisms [18]. The maxillary arch is more affected than the mandibular one, with the maxillary canines and lateral incisors being the most affected teeth and the central incisors the least [16,17,20]. The posterior teeth were the most affected on the mandibular arch [16].

### 3.2. Refractive Index

The optical properties of teeth, such as colour, translucency and opacity, are strongly influenced by the structure and composition of dental tissue. The structure of enamel consists of 96% hydroxyapatite and 4% organic matter [21]. Hydroxyapatite and healthy enamel have the same refractive index (RI = 1.60–1.66), with RI being an important optical parameter of dental tissue [22]. 

When a WSL occurs, it signifies the demineralization of the enamel. The normal structure of hydroxyapatite is affected by the acidogenic attack, and in consequence, the RI changes. The WSL appears as a matte and opaque white spot due to light scattering at the interface between sound enamel and lesion [18,19]. When hydrated with saliva, the WSL has an RI of 1.33, and if dried, it has an RI of 1.00, the lesion being more visible when dried.

### 3.3. Treatment with Resin

The treatment of WSLs should accomplish the arrest of the carious development, reinforcement of porous lesion and reestablishment of the optical properties of the enamel all at the same time. 

Different methods of treatment have been tested over the years, such as fluoridation and the use of casein–phosphopeptide amorphous calcium phosphate for remineralization microabrasion to restore the optical properties of the enamel. These treatments accomplish one of the three demands to the detriment of the other two. Classical restoration techniques accomplish all the demands but are too invasive and too much healthy dental structure is lost. 

Resin infiltration was developed initially to arrest the progression of proximal caries [19,23]. It is a microinvasive technique proven satisfactory in situ and in vivo [19]. Today, it is used for both vestibular and proximal WSL. The optical properties post-treatment are qualitative due to the RI of the resin used. Depending on the composition of the resin, the RI varies from 1.47 to 1.52. As Oivanen et al. show in their study, different co-monomer mixtures influence the RI of the resin, BisGMA favouring an increased RI and a better chameleon effect [24]. The principle behind this technique is to infiltrate the porous lesion, thus arresting the carious lesion, strengthening the dental structure and improving aesthetics.

Both carious activity and depth influence the treatment outcome. Active carious lesions have bigger pores and a demineralized matrix; therefore, the resin can easily infiltrate. Inactive lesions are harder to penetrate due to the mineralized outer surface. Neuhaus et al. demonstrated in their study that the average penetration depth is 500 μm. A lower penetration depth is usually due to inadequate use of the resin [25].

### 3.4. Infiltration Technique

The technique demands the use of a rubber dam and a prior professional dental cleaning [23]. A 15% HCl etcher is used on the surface of the WSL for a total of 2 min, stirring from time to time with a microbrush. 15% hydrochloric acid is preferred to 37% phosphoric acid due to better removal of the surface layer of the lesion and a more in-depth penetration [26]. Afterwards, the etcher is washed off for 30 s and dried with the air spray. 

Then, 99% ethanol is used for 30 s to ensure a completely dry lesion. This promotes a better penetration of the hydrophobic resin into the lesion. This step is based on the premise that ethanol desiccates the collagen structure; therefore, the resin can better infiltrate the enamel matrix [26]. A visual inspection will assess the result; if the lesion appears white, matte and opaque, the etching was successful, and the infiltration can proceed; if not, the etching and drying steps should be repeated until the aspect of the lesion is satisfactory [1].

The resin is applied to the lesion surface and left to penetrate for 3 to 5 min, depending on the depth and area of the lesion. The excess resin must be removed with a cotton ball or dental floss [26,27]. Subsequently, the resin is photocured from 3 different angles for 40 s to ensure polymerization [27]. This process should be repeated once, but the second time, the resin should be left to penetrate for only 1 min. This step ensures that the shrinkage with the first polymerization is reinforced with a second layer of resin [26]. After the second light-curing, the tooth surface is polished.

### 3.5. Clinical Studies Results

According to Table 2, most researchers prove that resin infiltration treatment (RIT) has the best clinical outcome, both structurally and aesthetically, out of all WSL treatments. All studies argue that immediate results are satisfactory. Different trials state that the treatment is stable for at least 24 months, with no changes visible to the naked eye. Gholami et al. demonstrated that the infiltration treatment was durable for a period of 6 months, results that are in line with other studies [28]. Knaup et al. proved, using quantitative light-induced fluorescence, that resin infiltration was stable throughout the 12-month period [29]. Xi Gu et al.’s trial aids this theory [30]. Knösel et al. found that between 24 and 45 months, some colour and lightness changes occurred, but they were insignificant and invisible clinically.

Split-mouth studies sustain that RIT has a better outcome than other treatments. All split-mouth trials reviewed argue that RIT has a better immediate outcome, whereas fluoride varnish (FV) or casein phosphopeptide–amorphous calcium phosphate (CPP-ACP) products showed improvement only at follow-ups. FV alone had a limited outcome [31], but light-cured FV showed better outcomes [32]. Kannan et al. demonstrated that light-cured FV had a surprising outcome, both immediately and at the 3- and 6-month follow-up. It is believed that the resin-like properties of the used product better penetrated the lesion. Although immediate results prefer RIT, the results after 6 months favoured the light-cured FV [32,33,34,35,36].

**Table 2:** Resin infiltration treatment trials. Only trials performed on post-orthodontic treatment patients who used the resin infiltration treatment at least were screened.

Researcher	Study Design	Treatment	Results	References
Xi Gu et al. (2019)	Split-mouth, randomized clinical trial	RIT and micro-abrasion	RIT had better immediate and long-term results. It decreased the lesion size and maintained aesthetics for a 12-month period.	[30]
Giray et al. (2018)	Randomized Clinical Trial	RIT and FV	Using DIAGNOdent, it was demonstrated that RIT had a superior outcome than the FV. The carious lesion was better assessed by the RIT, and FV penetrated only the superficial layer of the WSLs.	[31]
Kannan et al. (2019)	Randomized controlled trial	RIT and light-cured FV	RIT had a better immediate outcome. After 6 months, the results were reversed.	[32]
Simon et al. (2022)	Randomized controlled trial	RIT and CPP-ACP	RIT had a better immediate outcome. CPP-ACP had significant results after one month and was stable through 12 months.	[33]
Gholami et al. (2023)	Clinical trial	RIT	Through spectrophotometry and digital photography, it was shown that RIT improved aesthetics and was durable for the duration of the study.	[28]
Knaup et al. (2023)	Clinical trial	RIT	RIT improved the appearance of WSLs and was stable for a 12-month period.	[29]
Knösel et al. (2019)	Second follow-up of split-mouth randomized controlled study	RIT	Some colour and lightness changes occurred between 24 and 45 months but are unlikely to be visible to the naked eye.	[36]

RIT: resin infiltration treatment; FV: fluoride varnish; CPP-ACP: Casein phosphopeptide-amorphous calcium phosphate; WSLs: white spot lesions.

To validate the superiority of RIT, other WSL treatments were reviewed (Table 3). The main course of treatment for WSLs, besides resin infiltration, is fluoridation or microabrasion. Microabrasion has a mechanical approach of removing the outer layer of the WSL, whereas fluoridation treats the lesion from within. Out of all the studied procedures, high-concentration fluoridated toothpaste was shown to be the most effective [37,38,39], but toothpaste with an increased fluoride percentage is debated by most clinicians. P11-4 is a self-assembling peptide that shows good results in remineralizing WSLs [36,40,41,42].

## 4. Discussion

White spot lesions are the deposition of fixed orthodontic treatments. They form in the prolonged presence of dental plaque, favoured by the difficulty of hygienization, and can be influenced by diet and genetic background. With the popular demand for dental aesthetics and minimally invasive treatments, resin infiltration techniques were developed. It is a short and simple in-office procedure with immediate results.

In the election of this treatment, the clinical aspect of the lesion, such as the area and depth, should be taken into consideration. Neuhaus et al. proved that smaller and superficial lesions have a better outcome than wider and deeper lesions [25]. His findings are in line with the results of other studies. Another characteristic to bear in mind is the duration of the lesion. The treatment is more potent in the case of early-stage active lesions [19]. Knösel et al. found that older lesions require more conditioning time [43]. In order to evaluate the treatment election, it is necessary to identify a specific appraisal method for the lesion.

Researchers have used a spectrophotometer, laser fluorescence tools and photographic software evaluation to assess the colour, fluorescence and surface area of WSLs [28,32].

Even if immediate results are satisfactory, long-term effects are an ongoing discussion. Knösel et al. implemented a split-mouth study design, reviewing 6-month results, stating that the durability of the treatment was adequate [43]. Paris et al. implemented the same study design, assessing the results of an 18-month period. The study consists of a radiographical evaluation of the lesion progression. Borderline significance was reached and was in accordance with other in situ studies. The progression of the carious lesion was significantly reduced compared to the untreated control [44]. Knösel et al.’s follow-up study proves that the aesthetics of infiltration treatment are durable for as long as 45 months [32]. Yetkiner et al. found that infiltration treatment is stable against discoloration [45].

Other studies have shown that discoloration may occur in patients with daily habits such as smoking or drinking coffee or red wine. Paolone et al. state that resin infiltration, while effective in treating white spot lesions, may lead to staining over time when exposed to food liquids and especially to smoke, potentially compromising the long-term aesthetic outcome of the restoration [46]. Leland et al. proved that unpolished resin-treated teeth have a significant increase in staining compared to polished teeth. They also suggest that red wine increases the probability of staining more than other food or liquids [47]. Sabti et al. have found that resin infiltration leads to a significant increase in staining compared to untreated enamel. These staining properties should be taken into account when making clinical decisions regarding the treatment of white spot lesions [48]. Saccucci et al. suggest that polishing the surface or bleaching treatment could reverse or treat the staining [49]. The patients should be informed of these possible side effects and negative outcomes. They should also be prompted to limit the use of red wine, coffee, black tea, cigarettes and generally foods with added food colouring [47,49].

It is proposed that the infiltrating resin should adhere to a set of requirements, including hydrophilic and anti-bacterial properties, low viscosity, a pleasing aspect, the capability of polymerizing into a solid state and most important, non-toxicity [46,50]. The polymerization capability might reinforce the demineralized enamel structure, which is already fragile [1,51]. Paris et al. studied the influence of the infiltrants on hardness and demineralization [52]. Bis-GMA-containing resins have lower polymerization shrinkage and better flexural strength than TEGDMA resins. However, due to the high viscosity of Bis-GMA-based resins, the enamel microhardness and demineralization resistance are not superior to that of TEGDMA resins; hence, the low viscosity and hydrophilic TEGDMA are preferred [52,53]. Chen et al. studied the effects of rapid aging on the mechanical properties of resin infiltration. His findings support those of Paris et al. and Tostes et al. TEGDMA resin is preferred, despite it having inferior properties to Bis-GMA products [54].

The advantages of this procedure exceed the disadvantages. Most commercial products are marketed towards aesthetical rehabilitation, which they successfully achieve. By filling the porous lesion, the carious progression and demineralization process is arrested [55], consequently decreasing the risk of secondary caries [7]. Resin infiltration delays traditional restorations, preserving the healthy enamel that borders the WSL without the risks of pulpal inflammation and sensitivity. In clinical trials, no patients reported pain or sensitivity after the resin infiltration technique, and no other observations were made [56]. The main disadvantages of this treatment are staining and the uncertainty of stability with time.

The limitations of this review include quantity and diversity of data, insufficient number of relevant reviews, clinical studies partial to the relative recency of the treatment and restriction of commercial product availability. Some methodology limitations of the clinical studies cited include sample size and unreliability of patients.

## 5. Conclusions

White spot lesions are a common unwanted outcome of orthodontic treatment that can be minimized with proper oral hygiene. Fluorosis and mineralization defects are other instances in which white spot lesions can be found. Infiltration with resins has an immediate satisfactory outcome. It has the advantage of being a quick and painless procedure. The resin that is used fills and permanently stabilizes the porous lesion, consequently arresting the carious progression and restoring aesthetics. This is a relatively new procedure, with the only negative outcome noted in the current literature is staining. Studies show that the treatment is stable for up to 45 months. More clinical studies are needed to evaluate the long-term duration and outcome of this treatment.

Although the resin infiltration treatment is a simple and painless procedure, not all practitioners and patients are informed about this minimally invasive treatment. Consequently, promoting this treatment is necessary, both in the medical field and general population. Moreover, taking into account the scarce number of clinical trials using resin infiltration, our future research perspective is to investigate the results of resin infiltration in different tooth structure pathologies.

## Figures and Tables

**Table 1:** Description of inclusion and exclusion process.

Level of Screening	Description of the Selection Process
First level of screening: database filters	Filters such as language, date of publication and article type were applied to the database search.
Second level of screening: title review	After the application of the filters above, the titles of the results were reviewed. Unclear sentencing or missing keywords in the title were reasons for exclusion.
Third level of screening: abstract review	Short, unspecific and vague abstracts were subject to dismissal.
Fourth level of screening: full article review	Only articles with a clear objective, valid methods and results were included in the research.

**Table 3:** Different white spot lesion treatments and their results.

Researcher	Study Design	Treatment	Results	Reference
Karabekiroğlu et al. (2017)	Randomized controlled study	CPP-ACP	CPP-ACP was not more effective than 1450 ppm fluoridated toothpaste; the researcher suggested another way of treatment.	[37]
Singh et al. (2016)	Clinical trial	1000 ppm fluoride toothpaste, FV, CPP-ACP	The fluoridated toothpaste had the better outcome. The use of varnish or CPP-ACP did not improve the outcome.	[38]
Hoffman et al. (2015)	Randomized controlled trial	Toothpaste containing NovaMin	No difference between NovaMin toothpaste and fluoride toothpaste. A trend of improvement was observed at the 3-month check-up, but no significant results.	[40]
Atteya et al. (2023)	Randomized controlled clinical trial	P11-4, NSF and NaF	P11-4 and NSF showed better management of the carious activity than the NaF.	[41]
Sedlakova Kondelova et al. (2020)	Randomized controlled, split-mouth study	P11-4, FV	P11-4 reduced the size of the WSLs; P11-4 and delayed FV showed improved aestethics.	[42]
Memarpour et al. (2015)	Randomized clinical trial	FV, CPP-ACP	FV and CPP-ACP showed improvement of WSLs in primary teeth.	[39]

CPP-ACP: Casein phosphopeptide-amorphous calcium phosphate; FV: fluoride varnish; P11-4: self-assembling peptide P11-4; NSF: nano silver fluoride; WSLs: white spot lesions.
